# Identification of amino acid residues involved in substrate specificity of plant acyl-ACP thioesterases using a bioinformatics-guided approach

**DOI:** 10.1186/1471-2229-7-1

**Published:** 2007-01-03

**Authors:** Kimberly M Mayer, John Shanklin

**Affiliations:** 1 Brookhaven National Laboratory, Department of Biology, Upton, NY 11973 USA; 2 University of North Carolina at Wilmington, Center for Marine Science, Wilmington, NC 28409 USA

## Abstract

**Background:**

The large amount of available sequence information for the plant acyl-ACP thioesterases (TEs) made it possible to use a bioinformatics-guided approach to identify amino acid residues involved in substrate specificity. The Conserved Property Difference Locator (CPDL) program allowed the identification of putative specificity-determining residues that differ between the FatA and FatB TE classes. Six of the FatA residue differences identified by CPDL were incorporated into the FatB-like parent via site-directed mutagenesis and the effect of each on TE activity was determined. Variants were expressed in *E. coli *strain K27 that allows determination of enzyme activity by GCMS analysis of fatty acids released into the medium.

**Results:**

Substitutions at four of the positions (74, 86, 141, and 174) changed substrate specificity to varying degrees while changes at the remaining two positions, 110 and 221, essentially inactivated the thioesterase. The effects of substitutions at positions 74, 141, and 174 (3-MUT) or 74, 86, 141, 174 (4-MUT) were not additive with respect to specificity.

**Conclusion:**

Four of six putative specificity determining positions in plant TEs, identified with the use of CPDL, were validated experimentally; a novel colorimetric screen that discriminates between active and inactive TEs is also presented.

## Background

Plant acyl-acyl carrier protein (ACP) thioesterases (TEs) hydrolyze acyl-ACP thioester bonds, releasing free fatty acids and ACP. Plant acyl-ACP TEs are nuclear encoded, plastid-targeted globular proteins [1] that are functional as dimers [2,3]. Their activity represents the terminal step in the plastidial fatty acid biosynthesis pathway. The resulting free fatty acids enter the cytosol where they are esterified to coenzyme A and further metabolized into membrane lipids and/or storage triacylglycerols.

Plant acyl-ACP TEs have characteristic chain length specificities that vary from 8–18 carbons, and the substrate preferences of individual TEs have been shown to play a key role in determining the composition of storage lipids [1,4,5]. Based on amino acid sequence alignments, the plant TEs have been shown to cluster into two families, FatAs, which show marked preference for 18:1-ACP with minor activity towards 18:0- and 16:0-ACPs; and FatBs, which hydrolyze primarily saturated acyl-ACPs with chain lengths that vary between 8–16 carbons [5-7]. FatAs and FatBs both contain predicted ~60 amino acid transit peptides, however, FatBs have an additional conserved hydrophobic 18-residue domain that can be removed without affecting activity and which has been proposed to form a helical transmembrane anchor [8]. With the exception of two short regions that are unique to each class, the FatA and FatB sequences contain a core region of ~210 residues that show dispersed sequence similarity throughout.

Because of their importance in determining which fatty acids are stored in seed oil, several studies have focused on engineering plant TEs with altered substrate specificities as a strategy for tailoring specialty seed oils [8]. These studies have taken advantage of the rich diversity of sequence information available for the plant thioesterases and used a sequence-based approach to engineering plant thioesterases with altered substrate specificity [5,8-10]. However, the large amount of sequence variation between the FatA and FatB types of plant TEs makes it difficult to determine which amino acid residues are particularly important for substrate specificity.

When the amount of sequence variation between groups is high, bioinformatics tools can guide the development of a hierarchy of amino acid residues potentially important for specificity. The likelihood of success of this approach for the plant acyl-ACP TEs is increased by the availability of the above mentioned sequence information as well as by a 3D structural model of the FatB enzyme [11]. Furthermore, to assist in such an approach we recently developed the computer program Conserved Property Difference Locator (CPDL) [12]. CPDL identifies positions in an alignment of two functional classes of homologous proteins where each class has a conserved but different amino acid residue. This type of position has been shown repeatedly to be involved in functional specialization and of use in engineering proteins to switch their function from one class to that of the other [8,9,13,14]. Once identified by CPDL, these residues can be targeted for reciprocal switches between the two groups to introduce variability and evaluate their effects on enzyme function. CPDL identified many positions in the thioesterase family that show differences in either sequence or amino acid properties between the FatA and FatB classes. We evaluated the effects of several of the most dramatic changes identified by CPDL on thioesterase activity and substrate specificity. Using this approach we were able to identify four positions which influence the substrate specificity of the enzyme.

## Results

### Description of the homologous classes

**Figure 1:**
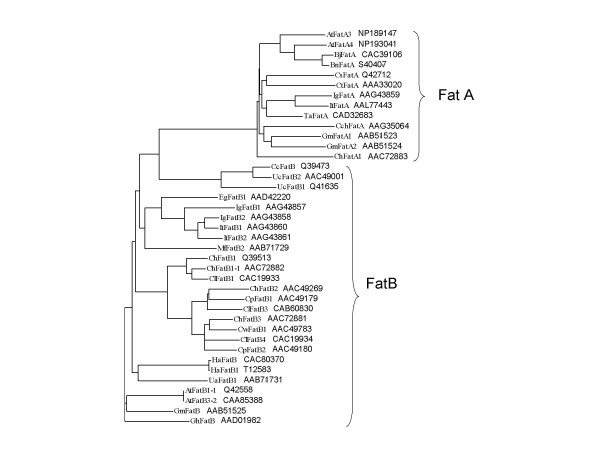
The FatA and FatB classes of plant acyl-ACP thioesterases. Enzymes in the FatA class are active on 18:1-ACP while those in the FatB class are active on saturated fatty acids of various chain lengths. Only enzymes whose substrate specificity has been demonstrated experimentally are shown. The NCBI accession numbers are provided in the figure. Numbers in the enzyme name were designated by depositors, except in the case of AtFatA3 and AtFatA4 where the number refers to the chromosome location of the gene as a way to distinguish between the sequences. At, *Arabidopsis thaliana*; Bj, *Bradyrhizobium japonicum*; Bn, *Brassica napus*; Cc, *Cinnamonum camphorum*; Cch, *Capsicum chinense*; Ch, *Cuphea hookeriana*; Cl, *Cuphea lanceolata*; Cp, *Cuphea palustris*; Cs, *Coriandrum sativum*; Ct, *Carthamus tinctorius*; Cw,*Cuphea wrightii*; Eg, *Elaeis guineensis*; Gh, *Gossypium hirsutum*; Gm, *Garcinia mangostana*; Ha, *Helianthus annuus*; Ig, *Iris germanica*; It, *Iris tectorum*; Mf, *Myristica fragrans*; Ta, *Triticum aestivum*; Ua, *Ulmus americana*; Uc, *Umbellularia californica*.

**Figure 2:**
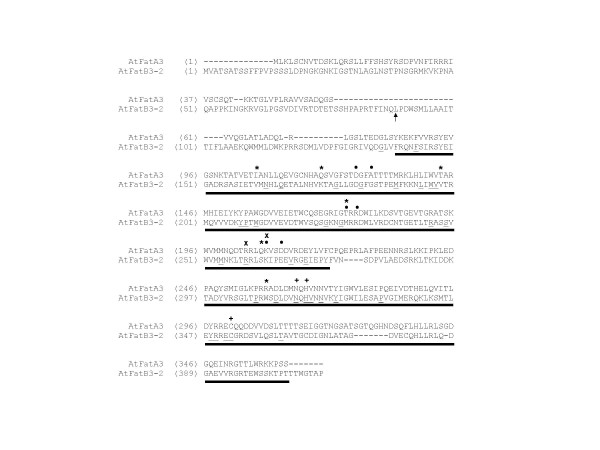
Amino acid sequence alignment of *Arabidopsis thaliana *FatA3 (NP_189147) with FatB3-2 (CAA85388). The first residue of the mature enzyme is marked with an arrow. The two hot-dog domains of the 3D structural model [11] are underlined. Completely conserved residues are underlined. Amino acid positions determined to be SDPs by previous authors are marked with filled circles. Asterisks denote the CPDL-identified putative specificity determining positions. + marks residues comprising the catalytic triad of C, H, N. X's mark positions where mutations inactivated the enzyme.

The two functional classes of plant acyl-ACP thioesterases (unsaturated fatty acid-recognizing Fat A versus saturated fatty acid-recognizing FatB) are well-defined in phylogenetic trees (Figure 1) constructed from multiple sequence alignment information (the .msf file is provided [see Additional file 1]). Each thioesterase contains a transit peptide of variable sequence at the N-terminus that signals their import into the chloroplast, thus only the mature protein sequences were considered in our analyses (starting at L88 in AtFatB). Overall, the family has relatively low sequence identity (10.4%), although identity is higher within classes (44.2% within the 13 FatA's, 19.7% within the 26 FatB's). Residues that are completely conserved within the family include N227, H229, and C264 (Figure 2), each of which has been implicated in catalysis and may form a papain-like catalytic triad [11,20].

### CPDL-identified positions

**Figure 3:**
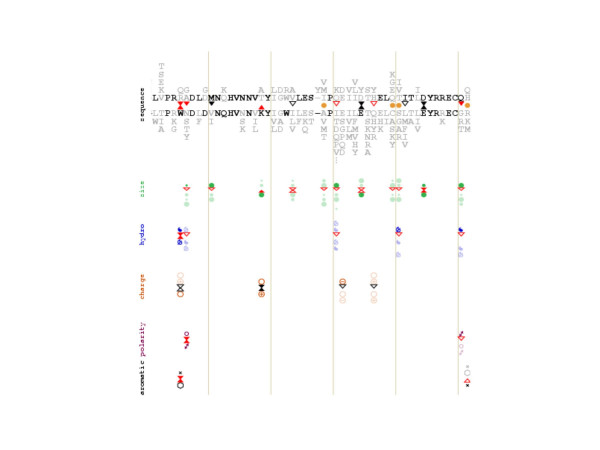
A portion of the CPDL [12] output for the FatA (upper) versus FatB (lower) alignment. Arrows denote residues that are conserved in all or "all-but-one" of the sequences in each class. One of the putative SDPs examined in this study (W221R) is flagged with a red hourglass.

Of the >400 positions within the thioesterase alignments, CPDL identified 67 positions within the family where there is conservation of sequence and 6 positions where there is conservation of amino acid properties within one of the classes (for example, see Figure 3). At nine positions, residues are conserved in each class but different between classes and are thus annotated with hourglasses (Table 2). Four of the nine hourglass positions are colored black, indicating conservative differences between the classes. The other five hourglasses are red and denote non-conservative differences between the classes. Many positions are marked with orange circles to denote property differences that are not necessarily accompanied by a consistent sequence difference. One such position is 86 where the FatB's always contain a charged residue (21 have lysine, 4 have arginine, 1 has glutamate) while the FatA's always contain the neutral glutamine.

Because they represent the most dramatic differences between the two thioesterase classes, we chose to evaluate the effect of each of the residues flagged with red hourglass icons. We also chose to examine the effect of position 86 as an example in which enzyme sequence is not conserved, but a particular property difference (in this case charged versus neutral) is conserved.

### In vivo thioesterase activity of CPDL variants

**Figure 4:**
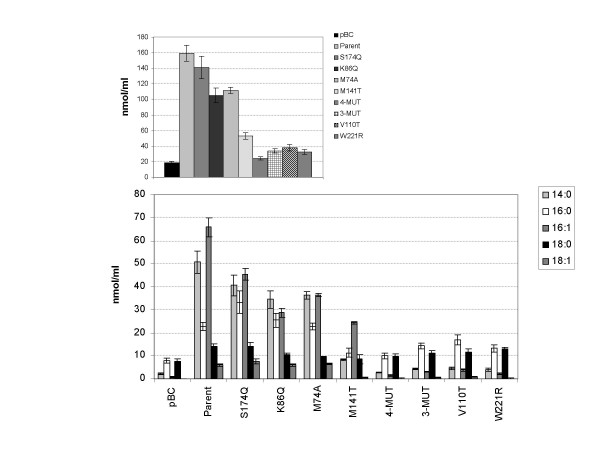
(A) The total fatty acid content (nmol/ml) in the medium from E. coli clones containing the variants listed as determined by GCMS of FAMEs. (B) Quantity of each fatty acid present in each of the variants. Error bars represent the standard error for five independent clones of each variant.

Each variant was evaluated in a bacterial expression system that allows determination of enzyme activity by measurement of the fatty acids present in the medium [7,9,10,19]. Individual colonies containing TE variants were grown in liquid medium at 30°C for 36 hr. The medium was collected and fatty acid methyl esters were prepared and then analyzed by GCMS. These assays showed that each mutation had an effect on thioesterase activity (Figure 4a). In particular, the V110T and W221R mutations were detrimental and had little detectable activity in the *in vivo E. coli *assay. While the M141T mutation lost ~75% total activity, (Figure 4a) some activity towards 16:1 was preferentially retained (Figure 4b). Furthermore, the amount of fatty acid in the medium represented by 16:1 for the M141T variant is ~70% as compared to ~46% in the parent. The M74A and K86Q mutations each decreased total activity slightly (Figure 4a) and showed a shift in specificity away from 14:0 and 16:1 (Figure 4b). The S174Q mutation resulted in only a slight decrease in total activity (Figure 4a), a decrease in both 14:0 and 16:1, and an increase in 16:0 and 18:1 as compared to the parent enzyme (Figure 4b). Two variants containing combinations of three (3-MUT: M74A, M141T, S174Q) or four (4-MUT: M74A, K86Q, M141T, S174Q) active mutations showed further reductions in activity (Figure 4a).

Because the M141T mutation substantially re-oriented specificity toward 16:1, we wanted to determine what effect other residues at this position might have on TE activity and specificity. Of the 84 saturation mutagenesis variants chosen for FAME analysis, none were found to have more 16:1 in the medium than the M141T variant (data not shown). Variants containing arginine, leucine, or isoleucine produced amounts of 16:1 similar to the threonine variant and were equally active while those containing glycine or phenylalanine were less active and produced less 16:1 than the threonine variant (data not shown). Sequencing of several active and inactive variants showed that the library contained at least 21 of the 32 possible codons at position 141. Of the 43 variants sequenced (representing 50% of the library), none had valine, glutamine, or histidine at position 141. All other amino acids were represented in the library.

### Agar-plate based screen for TE activity

**Figure 5:**
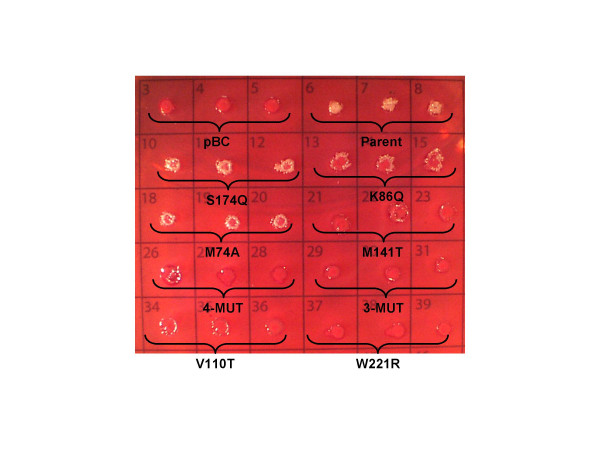
MacConkey agar plate-based screen for plant thioesterase activity. Colonies that contain an active thioesterase variant are white while those containing either empty vector (pBC) or an inactive variant are dark pink. Colonies exhibiting a range of activities could be reliably screened visually with this assay.

When plated on BTNA agar, there are subtle changes in colony morphology between variants expressing active plant acyl-ACP TEs and those expressing inactive TEs ([10] and KMM personal observation). However, we hoped to identify a more dramatic difference in order to facilitate future screening of libraries of variant TEs. Reasoning that the fatty acids eliminated into the growth medium by *E. coli *strain K27 would decrease the local pH around colonies that were expressing active variants of the plant acyl-ACP TE, we set out to find a pH indicator that could be added to agar plates for screening purposes. We were able to reproducibly screen for TE activity using standard MacConkey agar with lactose as the sugar source; the pH indicator neutral red changes from red (pH 6.8) to yellow (pH 8.0). A range of intensity in the red color is apparent when evaluating colonies that express TEs with a range of activities (Figure 5). However, the color of the variants expressing an active plant TE is white not red, as would be expected if the color change were being caused by the lower pH due to the excreted fatty acid. Thus, instead of reflecting a change in the local pH around active TE variants, the neutral red indicator may actually reflect a difference in the composition and stability of the bacterial membrane. For example, the ability to take up neutral red from the medium has been linked to virulence in *Mycobacterium tuberculosis* and has been shown to be the result of changes in the fatty acid composition and external surface of the cell membrane [21].

## Discussion

The modification of thioesterase specificity has proven to be useful for genetic engineering of plants containing high levels of commercially-useful fatty acids. For example, expression of a thioesterase from the California Bay Laurel (*Umbellularia californica*) in canola allowed the commercial production of a genetically engineered oil crop containing large amounts of laurate [5] while expression of a thioesterase from *Garcinia mangostana *in canola resulted in seeds containing increased amounts of stearate [22].

Using an approach that compares the sequences of homologous enzymes with different substrate specificities, the substrate specificity of plant thioesterases has been shown to be mutable. However, the large number of amino acid differences between any two homologous TEs makes it difficult to identify the subset of amino acid changes that will result in a change in specificity. One commonly used approach to reduce the number of possible SDPs is to generate chimeric enzymes [9]. Using this approach, it was found that the normally high 12:0 specificity of the *Umbellularia californica *FatB enzyme can be switched to 14:0 by three amino acid changes (M197R/R199H/T231K) [9]. However, oftentimes the resulting chimeric enzymes are either inactive or exhibit no change in specificity [8,23]. What would be helpful is a method that allows the reduction of the possible SDPs to a manageable, ranked set where each change can be individually examined experimentally.

We previously reported on the Conserved Property Difference Locator (CPDL) which was designed for use in such situations [12]. CPDL uses as input the amino acid sequence alignment of a group of enzymes broken into two homologous classes and then flags positions where there is a difference in either amino acid sequence or a property such as hydrophobicity [12]. From the alignment of FatA versus FatB TEs, CPDL identified several potential specificity-determining positions. We chose to use the most stringent CPDL criteria and therefore individually engineered into the parent enzyme the six most dramatic changes, including five non-conservative changes and one position with a difference in amino acid charge between FatAs and FatBs.

Interestingly, four of the five residues flagged with red hourglasses identified by CPDL as putative specificity-determining positions (74, 110, 141, 174) are located in a structural element referred to as the N-terminal hot dog domain [11]. Through the construction of chimeric enzymes, this region has been shown to control specificity [9]. The remaining position flagged by a red hourglass (221) is near the catalytic asparagine and histidine in the second hot dog domain. However, only one of the four residues flagged with black (conservative) hourglasses identified by CPDL is in the N-terminal hot dog domain, lending validity to the selection of sites that contain conservative versus non-conservative substitutions between classes as a criterion for ranking putative specificity determining positions.

Each of these six changes suggested by CPDL were individually engineered into the parent FatB enzyme and the effect of the change was determined experimentally. Mutations at each CPDL-identified position substantially affected thioesterase activity and/or specificity. Two of the six (V110T and W221R) essentially inactivated the enzyme while the other four mutations affected substrate specificity to some degree. It is interesting to note that unlike previous studies [9], combinations of mutations at multiple CPDL-identified positions (variants 3-MUT and 4-MUT) did not improve enzymatic performance and in fact, came close to eliminating activity.

**Figure 6:**
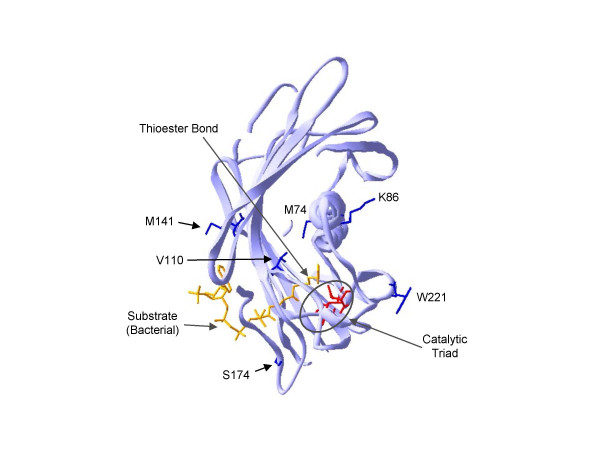
3D structural model of the AtFatB enzyme [11]. The CPDL-identified residues are shown in blue. The catalytic triad is circled with the residues colored red. The substrate from the bacterial enzyme is shown in orange for reference.

We recently modeled the predicted structure of the plant acyl-ACP thioesterases [11]. Using this model, we mapped the CPDL mutations relative to the predicted active site of the thioesterase (Figure 6). Each of the positions is located within 16 Angstroms of the nearest catalytic residue. The monomer of the enzyme itself in the structural model is ~43 × ~49 × ~35 Angstroms. Mutations at the two positions farthest away from the catalytic site (141 and 86) have been shown to affect enzyme specificity (here and [9]). Mutations at the two positions closest to the catalytic site (each ~8 Angstroms away) essentially inactivated the enzyme (110 and 221). Position 141 shifted specificity toward 16:1 *in vivo *and is located on one of the b-sheets. Position 184, which caused a shift in specificity from 14:0 and 16:1 toward 16:0 and 18:1, is located 11 Angstroms from its closest catalytic neighbor and is on a flexible loop that has the potential to shift during binding and/or catalysis. Because this is a flexible region, there is some uncertainty regarding its conformation in the FatB structure relative to the FatB model based on threading onto the 1BVQ structure [11].

Many characteristic properties of the amino acid residues present at the CPDL-identified positions are also different between the classes. To summarize these changes, the alanine is smaller than the methionine at position 74, the threonine is smaller than methionine and has an OH group at position 141, the lysine to glutamine change at position 86 removes a positive charge and adds an amine group, the serine to glutamine change at position 174 removes an OH and adds and amine, the valine to threonine change at position 110 adds an OH, and the tryptophan to arginine change at position 221 removes a bulky aromatic side chain and adds a positive charge. The net affect of these changes appears to be a widening of the substrate binding pocket in FatA as compared to FatB (see Figure 6).

The results presented here further demonstrate the viability of a sequence based approach as opposed to a more time consuming and complicated approach based on x-ray crystallography. Development of the CPDL tool facilitated a sequence-based bioinformatics approach to engineering plant acyl-ACP thioesterases for alterations in substrate specificity. Furthermore, CPDL analysis provides a straightforward method for generating hypotheses that can readily be tested regarding specificity determining positions within enzymes.

## Conclusion

Based on comparison of families of FatA and FatB TE sequences the CPDL program was used to identify six putative specificity determining positions. Substitutions of FatA equivalents into FatB resulted in changes in specificity at four of the positions validating the *in silico *CPDL predictions. In addition, a novel colorimetric screen able to discriminate between the expression of active and inactive TEs is presented.

## Methods

### CPDL analysis of plant acyl-ACP thioesterases

All sequences were obtained from NCBI and accession numbers are provided in Figure 1. Only enzymes whose substrate specificity has been demonstrated experimentally were included in the phylogenetic analysis. Amino acid sequences were aligned using CLUSTALW (v 1.82) with default parameters [15] and the subsequent phylogenetic analyses were done using PHYLIP with default parameters [16]. TREEVIEW [17] was used to display the resulting trees. The CPDL [12] program settings were adjusted to flag positions that are conserved in either group but different between groups in either amino acid sequence or any of five residue properties (including size, hydrophobicity, charge, polarity, and aromaticity). Analysis of the CPDL-identified residues in context in the predicted 3D model of Arabidopsis FatB (PDB id: 1XXY; [11]) was performed using DeepView [18].

### Cloning and *E. coli* expression system

The coding sequence of the mature AtFatB was amplified from plasmid TE3-2 [19] with primers FatBF (Table 1) and FatBR and cloned into the pBC expression plasmid [10] using the XhoI and SpeI restriction sites. The final plasmid construct pBC(AtFatB-par) contains three amino acid residue differences (I176L, E178D, L202S) as compared to the NCBI sequence (accession # Z36911). Each of the CPDL variants was constructed by overlap extension PCR using AtFatB-par as template in combination with the primers listed in Table 1 then cloned into the pBC expression plasmid. At each relevant position, the most common residue from AtFatA was introduced into AtFatB-par.

Saturation mutagenesis was performed at position 141 via PCR using either the FatBF and MSatR primers (reaction 1) or the MSatF and FatBR primers (reaction 2). Each reaction contained 10 mM of each primer, 10 mM dNTPs, 1 U Pfu DNA polymerase (Stratagene), and 15 mM MgCl_2 _in PCR buffer (100 mM Tris, 250 mM KCl, pH 8.3). Thirty cycles of 94°C for 30 sec, 45°C for 30 sec, and 72°C for 60 sec were performed. The fragments were gel-purified (Zymo Research) and then combined to use as template in an overlap extension PCR with the FatBF and FatBR primers. Each reaction contained 10 mM of each primer, 10 mM dNTPs, 1 U Advantage cDNA Taq polymerase (Clontech), and 35 mM MgCl_2 _in PCR buffer (100 mM Tris, 250 mM KCl, pH 9.2). Thirty cycles of 94°C for 30 sec, 40°C for 30 sec, and 72°C for 90 sec were performed. The ends of the resulting ~1.5 kb band were cut with XhoI and SpeI (New England Biolabs) and then the band was gel-purified (Zymo Research) before ligating the fragment into the pBC plasmid. The ligation mixture was used to transform chemically-competent K27 cells. The transformation mixture was spread on LB plates containing chloramphenicol and placed at 30°C overnight. Eighty-four colonies were picked into a 96-well plate containing 600 ml of BTNA medium (10 g NZ-amine and 5 g NaCl per L, pH 7.0) containing chloramphenicol. Four colonies each of K27 with pBC (empty vector control) and parent (positive control) were included on the same 96-well plate.

For fatty acid analysis, each pBC-based plasmid was transformed into the K27 strain of *E. coli *(CGSC5478). Strain K27 contains a mutation in the FadD enzyme of fatty acid biosynthesis that prevents uptake of free fatty acid from the medium. Thus, when an acyl-ACP thioesterase is expressed in this system, the free fatty acid product of the thioesterase reaction is secreted to the medium and remains there [9]. Transformed cells containing any of the plasmid constructs were grown at 30°C on BTNA medium containing 170 mg/ml chloramphenicol. Five colonies of each variant were grown individually for fatty acid analysis.

### Fatty acid analysis

Fatty acid content of the medium from various cell cultures was determined by the production and measurement of fatty acid methyl esters. Briefly, 22 μl of glacial acetic acid and 1 ml of 1:1 (vol:vol) chloroform:methanol was added to 0.5 ml of medium from pelleted cells corrected to give equivalent cell density based on A550. After mixing by inversion, the phases were separated by centrifugation and the lower phase was transferred to a fresh glass tube. The chloroform was evaporated by N_2 _stream, 1 ml of 2% H_2_SO_4 _in methanol was added, and the samples were heated to 90°C for 1 h. Samples were extracted once with 1 ml of 0.9% NaCl and 2 ml of hexane. The organic phase was transferred to a fresh tube and dried under N_2 _and then resuspended in 50 μl of hexane. 3 μl samples were analyzed on a Hewlett-Packard 6890 gas chromatograph equipped with a 5973 mass selective detector (GC/MS) and a J&W DB-23 capillary column (60 m × 250 μm × 0.25 μm). The injector was held at 225°C, the oven temperature was varied (100–160°C at 25°C/min, then 10°C/min to 240°C), and a helium flow of 1.1 ml/min was maintained. FAMEs were prepared individually from five colonies of each variant, as well as the parent and pBC-containing clones.

### Agar-plate screen for TE activity

To screen for active plant TE variants, colonies were plated on MacConkey agar (Sigma, St. Louis, MO) containing 170 mg/ml chloramphenicol. After growth overnight at 30°C, colonies containing active TE variants are white while those containing inactive TE variants are pink.

## Abbreviations

ACP, acyl carrier protein; CPDL, Conserved Property Difference Locator; FAME, fatty acid methyl ester; GCMS, gas chromatography mass spectrometry; PCR, polymerase chain reaction; SDP, specificity determining position; TE, thioesterase

## Authors' contributions

JS and KMM conceived and designed the experiments. KMM carried out the experiments. KMM and JS analyzed the data and drafted the manuscript. Both authors have read and approved the manuscript.

## Supplementary Material

Additional file 1 TE Multiple Sequence Alignment. This is an amino acid sequence alignment of FatAs and FatBs using CLUSTAL W. Click here for file

## Figures and Tables

**Table 1:** Sequences of primers used in this study.

Primer Name	Sequence (5'-3')
MAF	ATAGAAACCGTCGCAAATCATCTGCAGG
MAR	CCTGCAGATGATTTGCGACGGTTTCTAT
KQF	TAATCATGTTCAGACTGCTGGATTGCTTGG
KQR	CCAAGCAATCCAGCAGTCTGAACATGATTA
VTF	GATATGGGTTACTACTCGTATGC
VTR	GCATACGAGTAGTAACCCATATC
MTF	GGAAAGAATGGTACTCGTCGTGATTGGCT
MTR	AGCCAATCACGACGAGTACCATTCTTTCC
SQF	TGACTCGCCGGCTGCAGAAGCTGCCGGAGGACGTG
SQR	CACGTCCTCCGGCAGCTTCTGCAGCCGGCGAGTAC
WRF	CTCACTCCTCGACGGAGTGACCTAGA
WRR	TCTAGGTCACTCCGTCGAGGAGTGAG
FatBF	GACTAGTTTACCTGACTGGAGCATGCTTCTTGC
FatBR	CGGCTCGAGGGTAGTAGCAGATATAGTT
MSatF	GGAAAGAATGGTNNSCGTCGTGATTGGCT
MSatR	AGCCAATCACGACGSNNACCATTCTTTCC

Modified nucleotides used to change amino acid residues are underlined.

**Table 2:** Residues identified by the CPDL program and flagged with a filled hourglass (black or red).

CPDL Flag Color	Residue (FatA vs FatB)
Black	S/A vs G (96)
	P vs T/S (209)
	Y/D vs E (249)
	D vs E (259)
Red	A vs M (74)
	T vs V/L (110)
	T vs M/R (141)
	E/Q vs S (174)
	Q/R vs W (221)
Orange	Q vs K (86)

Number given is residue position in mature AtFatB. Position 86 is flagged with a red triangle and orange circle to denote that Q (neutral) is conserved in the FatA's whereas most FatB's contain K (charged).
